# Three-dimensional structural analysis of eukaryotic flagella/cilia by electron cryo-tomography

**DOI:** 10.1107/S0909049510036812

**Published:** 2010-11-05

**Authors:** Khanh Huy Bui, Gaia Pigino, Takashi Ishikawa

**Affiliations:** a Paul Scherrer Institute, 5232 Villigen PSI, Switzerland; b ETH Zurich, Switzerland

**Keywords:** dynein, flagella, axoneme, tomography, cryo-EM

## Abstract

Electron cryo-tomography is a potential approach to analyzing the three-dimensional conformation of frozen hydrated biological macromolecules using electron microscopy. Since projections of each individual object illuminated from different orientations are merged, electron tomography is capable of structural analysis of such heterogeneous environments as *in vivo* or with polymorphism, although radiation damage and the missing wedge are severe problems. Here, recent results on the structure of eukaryotic flagella, which is an ATP-driven bending organelle, from green algae *Chlamydomonas* are presented. Tomographic analysis reveals asymmetric molecular arrangements, especially that of the dynein motor proteins, in flagella, giving insight into the mechanism of planar asymmetric bending motion. Methodological challenges to obtaining higher-resolution structures from this technique are also discussed.

## Abstract 2

Based on the molecular architecture revealed by electron cryo-tomography, the mechanism of the bending motion of eukaryotic flagella/cilia is discussed.

## 1. Introduction

**Figure 1:**
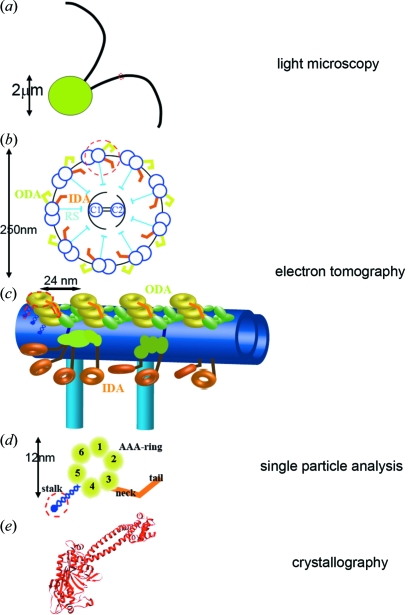
Structure of a flagellum and its components in various dimensions. The appropriate method for structural analysis at each scale is shown on the right. (*a*) *Chlamydomonas* cell with two flagella (5–10 µm length, 0.25 µm diameter). (*b*) Cross section [at the red dotted circle in (*a*)] of a flagellum. ODA: outer dynein arms. IDA: inner dynein arms. RS: radial spokes. (*c*) One microtubule doublet is extracted [red dotted circle in (*b*)], rotated and enlarged. (*d*) Schematic diagram of one dynein heavy chain [enclosed by the red dotted line in (*c*)]. (*e*) Atomic structure of the microtubule binding domain at the tip of the coiled-coil stalk.

Flagella and cilia are bending organelles in eukaryotic cells and are responsible for cellular motility or the flow of extracellular fluid (Fig. 1*a* ▶). The axoneme, the bending core of flagella and cilia, has a ‘9 + 2’ structure, where nine microtubule doublets surround two singlet microtubules. Adjacent microtubule doublets are connected by dynein heavy chains (ATP-driven motor proteins) and other linkers to form a sliding motion (Fig. 1*b* ▶). Dynein motor proteins form two complexes, the inner and outer dynein arms, responsible for waveform determination and force generation, respectively. Inner dynein arms contain eight dynein heavy chains and form the 96 nm periodicity along microtubule doublets, whereas outer dynein arms have two or three heavy chains with 24 nm periodicity (Fig. 1*c* ▶). Each dynein heavy chain (total 4500 amino acids) has an N-terminal tail (1500 amino acids), six AAA rings and a coiled-coil stalk (between the fourth and the fifth AAA domains) (Fig. 1*d* ▶). The N-terminal tail is anchored on one microtubule (A-MT), while the coiled-coil stalk dynamically interacts with the adjacent microtubule (B-MT) in an ATP-dependent manner.

Although the basic architecture of this ‘9 + 2’ axoneme is shared among most of the species, the waveform of the flagella shows amazing diversity depending on the species, organelles and conditions. For example, sperm flagella generate a symmetric waveform, while the waveform of *Tetrahymena* cilia is asymmetric. Normally *Chlamydomonas* cells swim toward the light with two flagella making a breast-stroke-like asymmetric waveform, whereas they swim backward with a sperm-like symmetric waveform under intense light. This conversion is regulated by calcium concentration. There should be a mechanism to integrate the sliding and bending of microtubule doublets driven by dynein into such a highly orchestrated flagellar/ciliary motion with well defined waveforms.

**Figure 2:**
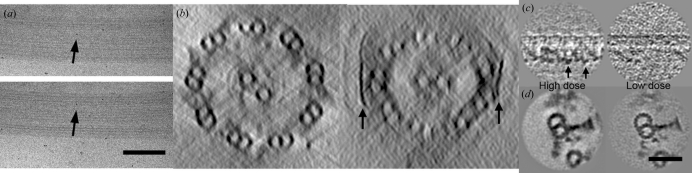
Effects of radiation damage and missing wedge artefacts on electron cryo-tomography images. (*a*) Two images from one series of tomographic acquisition. Top: ice-embedded flagellum tilted by 30°. Bottom: the same flagella without tilt. A gold label is shown by arrows. (*b*) Cross section of *Chlamydomonas* flagella with the membrane removed (left) and intact (right). The membrane is shown by arrows. Missing wedge artefacts generate non-isotropic density distributions. (*c*) Longitudinal (parallel to the microtubule) sections of averaged tomograms (ten particles) along microtubule doublets with high and low doses of electron beam. The averaged image with a high electron dose shows individual dynein molecules (arrows) even with ten particles. (*d*) Vertical sections of averaged tomograms (∼1000 particles) with high and low doses. By averaging many particles obtained under a low-dose condition better resolution is obtained. Averaging among nine doublets generates isotropic microtubules (even and round-shaped). (*a*) Scale bar = 250 nm. (*b*)–(*d*) Scale bar = 50 nm.

Despite previous two-dimensional image analysis of dynein heavy chains *in vitro* (Burgess *et al.*, 2003 ▶; Ueno *et al.*, 2008 ▶), the mechanisms of the power stroke of dynein and flagellar/ciliary bending motion were not known. To address this, three-dimensional structural analysis of dynein arms in flagella/cilia is required. NMR (Benashski *et al.*, 1999 ▶) and X-ray crystallography (Carter *et al.*, 2009 ▶) successfully solved the structure of a light chain and of a microtubule-binding domain of the dynein heavy chain (Fig. 1*e* ▶), respectively. Nevertheless, the full-length dynein heavy chain is still a very challenging target for such methods, and mainly analyzed by single-particle analysis from electron micrographs (Samso & Koonce, 2004 ▶). However, single-particle analysis, which requires the assumption that particles in micrographs are projections of an identical three-dimensional structure seen from random orientations, is not suitable for visualization of molecules in such a heterogeneous environment as flagella, where density from dyneins is overlapped with other molecules and dynein itself has heterogeneity owing to flexible domains. For this purpose, electron tomography is a potential approach to obtain the three-dimensional molecular structure *in situ* despite the limitation of resolution owing to radiation damage and the missing wedge described in the following two sections. In this research we acquired electron micrographs of ice-embedded flagella (thickness ∼0.3 µm), enabling us to observe the intact structure without artefacts from staining, fixation and flattening on carbon grids, tilted from −60 to +60° with a 2° increment angle using a CCD camera at a very low (∼0.5 e^−^ Å^−2^) electron dose (Ishikawa *et al.*, 2007 ▶). Acquired images were aligned using colloidal gold particles (Fig. 2*a* ▶).

Here we describe our recent *in situ* structural analysis of dynein and other proteins in flagella using electron cryo-tomography and propose a mechanism for flagellar/ciliary motion and its regulation of the waveform.

## 2. Electron dose and radiation damage for electron cryo-tomography

Frozen hydrated biological specimens (embedded in amorphous ice) have low contrast (Fig. 2*b* ▶) and high sensitivity to the electron beam. This is particularly important in the case of tomography, since the specimen must be illuminated multiple times and radiation damage is more severe than in other methods. To improve the signal-to-noise ratio, we utilized the periodicity of flagella (24 nm and 96 nm for outer and inner dynein arms, respectively) by averaging these periodic units, first along each microtubule doublet (Fig. 2*c* ▶) and then among nine doublets (Fig. 2*d* ▶). However, to extract fragments with periodicity and align them for averaging, contrast is required. More illumination gives higher contrast, but radiation damage suppresses the resolution. We must therefore find the optimized conditions to obtain sufficient contrast and sufficient resolution to address our requirements.

We acquired datasets at relatively low dose conditions (∼30e^−^Å^−2^ in total) to reveal the three-dimensional conformation of dynein heavy chains (described in §4), and relatively high dose conditions (∼60 e^−^ Å^−2^) when we map the arrangement of proteins in flagella to determine asymmetry (see §5). The three-dimensional reconstruction from images acquired under low-dose conditions shows less contrast (Fig. 2*c* ▶), but, after averaging, presents higher resolution than individual images under the high-dose condition (Fig. 2*d* ▶).

## 3. Missing wedge

Another problem with electron tomography is the missing wedge. Owing to technical problems (highly tilted samples are very thick owing to the projection at those angles and do not allow the electron beam to fully penetrate), the specimen can be tilted to only a limited angle (in our case 60°). Since one transmission electron micrograph, which is a projection of the object in real space, corresponds to a section in three-dimensional Fourier space, practical tomographic datasets can cover only a limited area and information is missing for wedge-shaped areas in three-dimensional Fourier space. This causes an artificial distortion of the three-dimensional reconstruction (Lucic *et al.*, 2005 ▶).

Indeed, in the vertical section of our tomogram of a flagellum, contrast of the microtubule doublets is weak in the direction perpendicular to the electron beam (*i.e.* horizontal), whereas the edges parallel to the beam (*i.e.* vertical) are not distorted (Fig. 2*a* ▶). The flagellar membrane is over-amplified in the vertical direction (arrows in Fig. 2*b* ▶) and vanishes in the horizontal direction. However, the problem is avoided by averaging nine microtubule doublets which are oriented differently and thus have different orientations of the missing wedge. After averaging, the cross section of a microtubule doublet shows an isotropic density distribution with each protofilament clearly visible (Fig. 2*d* ▶).

## 4. Conformation of outer and inner dynein arms in flagella

**Figure 3:**
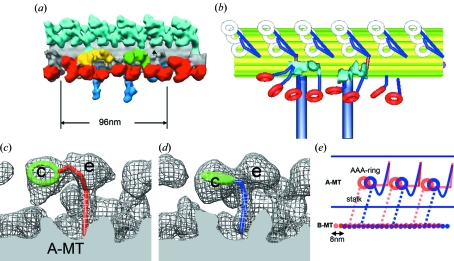
Dynein conformation in flagella. (*a*) Averaged tomogram utilizing 96 nm periodicity. Light blue: outer dynein arms. Red: inner dynein arms. Grey: microtubule doublet. (*b*) Molecular arrangement of dynein isoforms revealed by tomographic analysis of wild-type and mutant *Chlamydomonas* flagella. Modified from Bui *et al.* (2009 ▶). (*c*) Structure of inner arm dynein c before the power stroke. (*d*) As for (*c*) but after the power stroke. In (*c*) and (*d*), dyneins c and e are labeled. (*e*) Schematic representation of the power stroke. Left: proximal (−) end. Right: distal (+) end. Modified from Movassagh *et al.* (2010 ▶).

Based on the above approach, we reconstructed *in situ* three-dimensional structures of periodic units containing a microtubule doublet, outer and inner dynein arms as well as other proteins in flagella from green algae *Chlamydomonas reinhardtii* (Fig. 3*a* ▶) (Ishikawa *et al.*, 2007 ▶; Bui *et al.*, 2008 ▶). Three outer arm dynein heavy chains stack on the A-MT vertically, whereas eight inner arm dyneins form a collinear array. Comparison of mutants lacking some isoforms of inner and outer arm dyneins with the wild-type enabled us to identify the locations of those isoforms and also the positions of AAA rings and N-terminal tails (Fig. 3*b* ▶). All the dyneins have N-terminal tails extending from the AAA ring toward the distal end (*i.e.* tip of the flagellum and plus-end of the microtubule), except the outer arm γ heavy chain whose conformation is not yet clear. This suggests that all the dyneins generate force in the same direction. However, orientations of AAA rings show variety. Some dyneins may generate torque to control waveforms.

Kinetics studies (Holzbaur & Johnson, 1989 ▶) proved that the power stroke of dynein occurs during product (ADP and/or phosphate) release. Therefore, to understand the structural basis of the power stroke, we compared the three-dimensional structure of the outer and inner dynein arms with and without nucleotides (Movassagh *et al.*, 2010 ▶). We trapped the dynein ATPase cycle at the ADP.Pi state by adding vanadate. Compared with the structure in the presence of ADP and vanadate, the positions of the AAA rings are shifted toward the distal end in the absence of nucleotides (apo) (Figs. 3*c* and 3*d* ▶). The orientation of the coiled-coil stalk is invariant during the power stroke. This result indicates that the AAA ring shifts (but does not rotate) to pull the adjacent B-MT during product release (Fig. 3*e* ▶).

## 5. Asymmetry of the dynein arrangement on nine microtubule doublets

**Figure 4:**
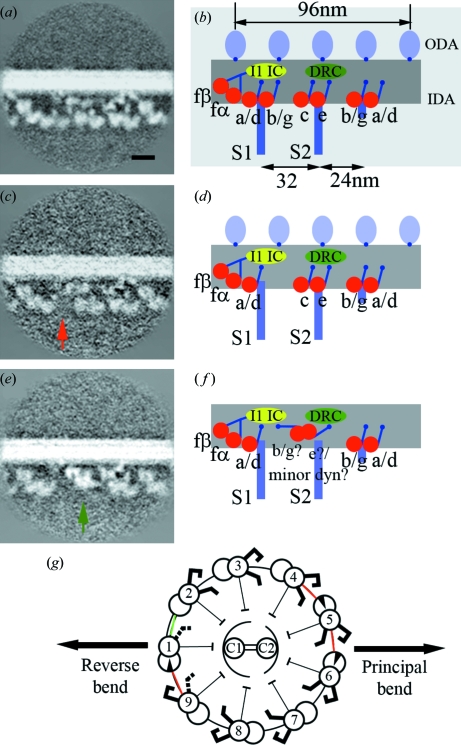
Asymmetry of the molecular arrangement of dynein and inter-doublet linkers in *Chlamydomonas* flagella. (*a*), (*b*) Common architecture seen in doublets 2–8 [defined in (*g*)]. (*c*), (*d*) Doublet 9. (*e*), (*f*) Doublet 1. (*a*), (*c*), (*e*) Density maps of averaged tomograms. Missing dyneins are shown by arrows. Scale bar = 20 nm. (*b*), *d*), (*f*) Models of the inner arm dynein arrangement. ODA: outer dynein arm. IDA: inner dynein arm. DRC: dynein regulatory complex. IC/LC: intermediate and light chains. S1, S2: radial spokes 1 and 2. Inner arm dyneins (a, b, c, d, e, f, g) are indicated. (*g*) Inter-doublet linkers are shown on a vertical section of a flagellum (green and red lines). Inner dynein arms lacking some isoforms are shown by black dotted lines. Modified from Bui *et al.* (2009 ▶).

It is still unclear how the sliding and bending motions of each pair of adjacent microtubule doublets are integrated into a well defined waveform. Electron cryo-tomography of *Chlamydomonas* flagella supplied us with an insight (Bui *et al.*, 2009 ▶) (Fig. 4 ▶). Under the conditions of relatively high electron dose, the differences between the architecture of dyneins among nine microtubule doublets were detected. Seven of nine doublets share an identical dynein arrangement. However, one doublet (doublet 9) lacks one inner arm dynein (dynein b/g) (Figs. 4*c* and 4*d* ▶). Furthermore, we realised an abnormality in the adjacent doublet (doublet 1). It lacks a few inner arm dyneins or they are mis-folded or replaced by other molecules (Figs. 4*e* and 4*f* ▶).

Asymmetry was detected in other proteins linking microtubule doublets (Fig. 4*g* ▶). Although nexin links all the adjacent pairs of microtubule doublets, we found two more linker proteins connecting only limited pairs of doublets: IDL2 exists between 4 and 5, 5 and 6, and 9 and 1, whereas IDL3 links doublets 1 and 2. These linkers are plotted almost in the plane of bending. They probably suppress the sliding motion between microtubule doublets, restricting the motion in the plane. Localized defects of the inner arm dyneins could make this planar bending asymmetric.

## 6. Discussion

Our results demonstrate the potential of electron cryo-tomography as a method for visualizing molecular arrangement, conformation and dynamics *in situ*. Current optics and detectors techniques limit the resolution of electron cryo-tomography to ∼35 Å. Even in this resolution range we could analyze the location and the conformation of dynein by utilizing mutants. Tagging techniques such as immunogold labeling and GFP fusion will be helpful as well. For image analysis at atomic resolution, we must combine the *in situ* structure from tomography with the high-resolution structure from crystallography, NMR or single-particle analysis.
